# *ZmFdC2* Encoding a Ferredoxin Protein With C-Terminus Extension Is Indispensable for Maize Growth

**DOI:** 10.3389/fpls.2021.646359

**Published:** 2021-04-23

**Authors:** Yue Chen, Deyi Zhong, Xiu Yang, Yonghui Zhao, Liping Dai, Dali Zeng, Quan Wang, Lei Gao, Shengben Li

**Affiliations:** ^1^Agricultural Genomics Institute, Chinese Academy of Agricultural Sciences, Shenzhen, China; ^2^College of Life Science, South China Agricultural University, Guangzhou, China; ^3^Plant Phenomics Research Center, Nanjing Agricultural University, Nanjing, China; ^4^State Key Laboratory of Rice Biology, China National Rice Research Institute, Chinese Academy of Agricultural Sciences, Hangzhou, China; ^5^College of Life Sciences and Oceanography, Shenzhen University, Shenzhen, China; ^6^State Key Laboratory of Crop Genetics and Germplasm Enhancement, Nanjing Agricultural University, Nanjing, China; ^7^Guangdong Provincial Key Laboratory for Plant Epigenetics, College of Life Sciences and Oceanography, Shenzhen University, Shenzhen, China

**Keywords:** *ZmFdC2*, electron transfer, photosynthesis, maize, growth

## Abstract

As important electron carriers, ferredoxin (Fd) proteins play important roles in photosynthesis, and the assimilation of CO_2_, nitrate, sulfate, and other metabolites. In addition to the well-studied Fds, plant genome encodes two Fd-like protein members named FdC1 and FdC2, which have extension regions at the C-terminus of the 2Fe-2S cluster. Mutation or overexpression of *FdC* genes caused alterations in photosynthetic electron transfer rate in rice and Arabidopsis. Maize genome contains one copy of each FdC gene. However, the functions of these genes have not been reported. In this study, we identified the *ZmFdC2* gene by forward genetics approach. Mutation of this gene causes impaired photosynthetic electron transport and collapsed chloroplasts. The mutant plant is seedling-lethal, indicating the indispensable function of *ZmFdC2* gene in maize development. The *ZmFdC2* gene is specifically expressed in photosynthetic tissues and induced by light treatment, and the encoded protein is localized on chloroplast, implying its specialized function in photosynthesis. Furthermore, *ZmFdC2* expression was detected in both mesophyll cells and bundle sheath cells, the two cell types specialized for C4 and C3 photosynthesis pathways in maize. Epigenomic analyses showed that *ZmFdC2* locus was enriched for active histone modifications. Our results demonstrate that ZmFdC2 is a key component of the photosynthesis pathway and is crucial for the development of maize.

## Introduction

Oxidation-reduction (Redox) reactions play central roles in cell metabolism, and electron transfer is one of the most important steps in these reactions. Cells evolved diverse electron carriers for distinct metabolic pathways. As a group of electron carriers widely utilized by prokaryotic and eukaryotic organisms, ferredoxins (Fds) belong to a conserved protein family. Plant genomes contain multiple Fd genes. According to the spatial specificity of their expression, Fd genes could be roughly divided into photosynthetic and non-photosynthetic categories. During plant photosynthesis, photosystem II (PSII) catalyzes water-split reaction to form high energy electrons, which are transmitted to the cytochrome (Cyt) b6f complex through plastoquinone (PQ) and accepted by photosystem I (PSI). PSI subsequently donates the electrons to Fds, the soluble electron acceptors in the chloroplast stroma (Joliot and Johnson, 2011). In linear electron flow (LEF), photosynthetic Fds subsequently donate the electrons to Fd-NADP^+^ oxidoreductase (FNR) to produce NADPH, the latter serves as the reducing power to assimilate CO_2_ and synthesize chlorophyll, fatty acids, some plant hormones and other metabolites in chloroplast (Hanke and Mulo, 2013). Alternatively, Fds could return the electrons to the plastoquinone (PQ) through cyclic electron flow (CEF). CEF generates more ATP without producing extra NADPH and is important for plant survival in different light environments (Yamori and Shikanai, 2016). In C4 plants, CEF in bundle sheath cell is the key process in producing extra ATPs required by phosphoenolpyruvate (PEP) regeneration (Yin and Struik, 2018). In addition, Fds expressed in roots have higher affinities with root FNR and provide reducing power for nitrite reductase, sulfite reductase and Glu synthase (Yonekura-Sakakibara et al., 2000; Flores et al., 2005).

Fds play pivotal roles in plant development. Knockdown or RNAi of *AtFd2*, the major ferredoxin gene in Arabidopsis leaves, dramatically reduced LEF and resulted in light green leaves and retarded growth (Hanke and Hase, 2008; Voss et al., 2008). Surprisingly, when plants were grown under long term and strong light conditions, the *AtFd2* knockout mutant displayed enhanced tolerance to high light stress (Liu et al., 2013). The different responses of *AtFd2* mutant to various light conditions imply the complicated regulation of plant photosynthesis by Fd proteins. Ectopically overexpressing *AtFd1* gene in tobacco led to enhanced CEF and inhibition of general photosynthetic electron transport (PET); the transgenic plants exhibited variegated leaves and retarded growth (Blanco et al., 2013). In addition, *AtFd2* expression was negatively responsive to pathogen treatment, and the *AtFd2* knockout mutant produced lower levels of reduced oxygen species (ROS) triggered by pathogen, leading to compromised pathogen resistance (Wang et al., 2018).

In addition to the well-studied Fd proteins, FdCs were identified recently and showed high similarities with Fds except for the extension regions at the C-terminus (Voss et al., 2011). FdC genes are widely spread and well-conserved across plant kingdom, however, their functions are largely unclear. According to the length of the C-terminus extensions, FdC proteins could be divided into FdC1 and FdC2 categories, of which FdC2 has longer extension relative to FdC1 (Guan et al., 2018). In Arabidopsis, *AtFdC1* is expressed in both photosynthetic and non-photosynthetic tissues. When the major PSI receptor gene, *AtFdII*, was mutated, *AtFdC1* expression was compensatively upregulated (Voss et al., 2011). AtFdC1 was able to interact with most PSI subunits and accept electrons from PSI, but interacted with photosynthetic FNR with very low affinity and failed to reduce NADP^+^, indicating its possible role in transmitting electrons for metabolic pathways such as nitrite and sulfite reduction, other than carbon fixation under PSI acceptor limitation (Guan et al., 2018). Although down-regulation by RNAi or overexpression of *AtFdC1* impaired photosynthetic electron transmit, both transgenic plants showed wild type-like phenotypes (Guan et al., 2018). Similarly, mutation of *OsFdC2* resulted in reduced photosynthetic rate. The plant displayed yellow leaves and delayed heading time, but was still viable and fertile (Li et al., 2015; Zhao et al., 2015). These results suggest the dispensable roles or functional redundancies of FdCs in Arabidopsis and rice.

As a typical C4 plant, maize assimilates CO_2_ in two different types of cells: mesophyll cells and bundle sheath cells. Maize genome encodes at least 9 predicted Fd proteins, among them, ZmFdI-VI were well-characterized on biochemical activities and expression profiles (Kimata and Hase, 1989; Hase et al., 1991; Matsumura et al., 1997). *ZmFd I* and *ZmFd II* are expressed in leaf mesophyll cells and sheath bundle cells, respectively, and their expressions can be induced by light, suggesting their functions in photosynthesis (Sakakibara, 2003). In contrast, *ZmFd III, IV*, and *VI* were preferentially expressed in root tissue and belong to non-photosynthetic ferredoxins. *ZmFdVI* was expressed in roots and quickly induced by nitrate, indicating its role in nitrate assimilation (Matsumura et al., 1997; Sakakibara, 2003). Genomic analysis showed that maize genome contains two FdC genes, *ZmFdC1* and *ZmFdC2*, however, neither of them have been studied in genetics or biochemistry, and their functions remain unknown.

In this study, we identified *ZmFd*C*2* gene in maize through mutant screen. *ZmFdC2* encodes a chloroplast localized protein and is specifically expressed in photosynthetic tissues and is upregulated by light, implying its function in photosynthetic electron transfer in maize. Genome wide analyses of epigenetic marks on this locus indicated that *ZmFdC2* expression may be under active regulation by histone modifications. Notably, *ZmFdC2* mutant is seedling-lethal, which is contrast to the wild type-like phenotype of the Arabidopsis *AtFdC1* mutant and the moderate effect on rice growth of *OsFdC2* mutation, implying more important roles of FdC2 in maize development.

## Materials and Methods

### Plant Material and Growth Conditions

The *pale and small1*(*pas1*) mutant was isolated from an EMS mutagenesis library conducted on the inbred line HZM. For plant growth, seeds were surface sterilized with 2% (v/v) sodium hypochlorite solution and seedlings were grown in a growth chamber at 30°C under 200 μmol m^−2^ s^−1^ intensity and 16 h light/8 h dark cycle. The youngest fully expanded leaves at the three-leaf stage were used for nearly all analyses unless otherwise noted.

### Chlorophyll Quantification

Chlorophyll contents were measured as previously described (Hartmut and Lichtenthaler, 1983; Wang et al., 2017). Briefly, 0.2 g fresh leaves of HZM and *pas1* plants were cut into pieces and incubated with 5 ml of 95% (v/v) ethanol at room temperature for 48 h in darkness. The slurry was centrifuged to remove the debris, and chlorophyll contents were determined at 655, 649 and 470 nm with Nanodrop 2000 spectrophotometer (Fisher Thermo Scientific). Chlorophyll a, b and x contents were calculated as Chla = 13.95 × D665-6.88 × D649, Chlb = 24.96 × D649-7.32 × D665, and Chlx = (1,000 × D470-2.05 × Chla-114.8 × Chlb)/245. Three biological replicates were determined for each sample.

### Transmission Electron Microscopy (TEM) Observation

The leaf fragments from one-week-old plants were fixed with 2.5% (v/v) glutaraldehyde in 0.1 M phosphate buffer (pH7.0) overnight followed by washing with phosphate buffer for 15 min three times. The samples were then subjected to post-fixing with 1% OsO4 in phosphate buffer for 2 h, and washed for 15 min with the phosphate buffer three times. The leaf fragments were subsequently dehydrated with a graded series of ethanol (30, 50, 70, 80, 90, 95, and 100% v/v) for about 15 min at each step followed by soaking with acetone for 20 min. The samples were then incubated with the 50 and 75% (w/w) Spurr resin (Sigma) in acetone for 1 h, and then moved to pure Spurr resin and incubated overnight. The resin was polymerized at 70°C. The specimen was sectioned with a LEICA EM UC7 ultratome and sections were stained by 98% (w/v) uranyl acetate for 5 min and 50% (w/v) alkaline lead citrate for 10 min subsequently. The chloroplasts were visualized with a HITACHI Transmission Electron Microscope (H-7650).

### Gene Mapping of *pale and small1 (pas1)*

Crosses were made between the *pas1* heterozygous plants and maize cultivar Q319 or Mo17. The *pas1* mutants could be easily distinguished from wild type-like plants based on the pale leaf phenotype. For map-based cloning, a total of 910 F_2_ homozygous mutant plants showing *pas1* phenotypes were used for DNA extraction. SSR and Indel markers were developed for gene mapping and their sequences are listed in Supplementary Table 1. The PCR procedure was as follows: 95°C for 5 min, followed by 34 cycles of 95°C for 30 s, 60°C for 30 s and 72°C for 30 s, and a final elongation step at 72°C for 5 min. PCR products were resolved with 4% agarose gels.

### Subcellular Localization of ZmFdC2

To construct plasmid for the subcellular localization of ZmFdC2, the coding sequence of *ZmFdC2* was amplified using specific primer pairs (listed in Supplementary Table 1) and inserted between the cauliflower mosaic virus (CaMV) 35S promoter and the GFP sequence of the *pBeacon-eGFP* vector. The plasmid was transiently expressed in maize protoplast and incubated in darkness at 28°C for 16 h. GFP fluorescence was visualized with LEICA SP8 confocal laser scanning microscopy. The emission wavelength was 488 nm. The excitation wavelength was 500–550 nm for GFP and 600–650 nm for chloroplast autofluorescence.

### Protein Interaction Assay

*ZmFdI, ZmFdII, ZmFdC2* and *ZmFdC2m* (*pas1*) were cloned into the bait vector *pGBKT7* between NcoI and BamHI; *ZmPsaE2, ZmLFNR1, ZmLFNR2, ZmNiR, ZmSiR, ZmFTRB, Zm-RFNR2, ZmPsaC, ZmPsaD, ZmPsaD2, ZmNdhS, ZmFTRA2, ZmFTRA1, ZmPGR5, ZmPGR5-L1*, and *ZmPGR5-L2* were obtained by BLAST against their homologs in Arabidopsis and cloned into the prey vector *pGADT7* by homologous recombination with pEASY-Basic Seamless Cloning and Assembly Kit (TransGen Biotech). Each pair of bait and prey plasmids were co-transformed into Y2HGold yeast strain (Clontech). The transforms were selected on DDO (SD/-Leu/-Trp) medium and the interactions were tested on TDO (SD/-Leu/-Trp/-His) and QDO (SD/-Leu/-Trp/-His/-Ade) medium.

### Protein Structure Simulation of ZmFdC2

The full-length peptides of ZmFdC2 and ZmFdC2m were analyzed with Phyre2 tool using spinach ferredoxin 1A70 as template (Kelley et al., 2015). The structures were visualized using the Jmol viewer.

### Quantitative RT-PCR Analysis

Total RNA was extracted with TRIzol™ Reagent (Thermo fisher scientific). Five hundred nanogram RNA was used for reverse transcription with ReverTra Ace qPCR RT Master Mix (TOYOBO). Real-time PCR was performed in three biological replicates with SYBR Green Mix (Vazyme) on ABI 7500 real-time PCR system. Primers used for RT-PCR are listed in Supplementary Table 1. The maize *Actin* gene was used as an internal control. The procedure was as follows: initial polymerase activation for 30 s at 95°C followed by 40 cycles of 95°C for 5 s and 60°C for 20 s. For each sample, qRT–PCR was performed with three biological replicates. The 2^−ΔΔCT^ method was used to analyze relative transcript levels in gene expression (Livak and Schmittgen, 2001).

### Chlorophyll Fluorescence Measurements

Maize plants were grown for 10 days in growth chamber at 30°C under 200 μmol m^−2^ s^−1^ intensity and 16 h light/8 h dark cycle. Chlorophyll fluorescence was measured on the third intact leaves (from bottom to top) with PAM-2500 chlorophyll fluorometer (Walz, Germany). Non-photochemical quenching (NPQ) was calculated as (Fm–F'm)/F'm); Photochemical quenching (qP) was calculated as (F'm–Fs)/(F'm–Fo); Linear electron transport rate (ETR) was calculated as [(F'm–F)/ F'm] × light intensity in μmol quanta m^−2^ s^−1^ × 0.5.

### Light Treatment on Maize

Maize seedlings were grown in dark at 30°C for a week in growth chamber, and were subsequently exposed to light at an intensity of about 200 μmol m^−2^ s^−1^ for 3, 9, or 24 h,. The youngest leaves were harvested from the treated plants of the wild type and *pas1* mutant for real time PCR.

### Nitrate Treatment on Maize

Maize seedlings were grown under conditions as previously described (Sakakibara, 2003). Briefly, the wild-type and *pas1* seedlings were grown in vermiculite with Hoagland's solution containing 1.6 mmol/L KNO_3_ for 2 weeks. For nitrate treatment, the seedlings were transferred to Hoagland's solution containing 16 mmol/L KNO_3_ and the youngest leaves were harvested after 1, 2, or 4 h treatment for real-time PCR. The plants were grown in a chamber at 30°C under 16 h light/8 h dark cycle.

### Separation of Mesophyll and Bundle Sheath Cell

The procedure followed the method in reference (Furumoto et al., 1999). Leaf blades from 1-week-old seedlings were cut into about 1 mm strips. The strips were immersed in 10 ml enzyme solution (1.5% cellulase R10 w/v, 0.3% macerozyme R10 w/v, 0.4 M mannitol, 20 mM KC1, 20 mM MES, 10 mM CaCl_2_, 0.1% BSA w/v, pH 5.7) by vacuum infiltration for 30 min and digested with gentle shaking at 25°C for 3 h. The digested mixture was filtered with 50 μm nylon filters. The mesophyll cells were pelleted from the filtered solution by 100 × ***g*** centrifugation followed by washing with cold washing solution (0.6 M mannitol, 10 mM MES, pH 5.7, 20 mM KC1) twice. The bundle sheath tissues were retained on the filter and extensively washed with the same wash solution. The bundle sheath tissues and mesophyll cells were examined with microscopy to ensure that there was no contamination from each other.

### RNA Sequencing (RNA-seq) Analysis

Total RNA was extracted from the youngest leaves of the seedlings at the three-leaf stage grown in growth chamber at 30°C under 16 h light/8 h dark cycle. mRNA was enriched from total RNA using oligo(dT) coated magnetic beads (Nanocs). cDNA was synthesized using random hexamer primers. Libraries were constructed using NEBNext Ultra RNA library prep kit (NEB) and 150 bp pair end RNA-sequencing was conducted with Illumina NovaSeq 6000 platform from the Annoroad Gene Technology company. Sequencing of each sample was performed for 3 biological replicates and the significance of differentially expressed genes (DEGs) was determined by fold change > 2 and *p*-values < 0.05.

## Results

### *pale* and *small1* (*pas1*) Mutant Is Defective in Chloroplast Development and Seedling-Lethal

**Figure 1:**
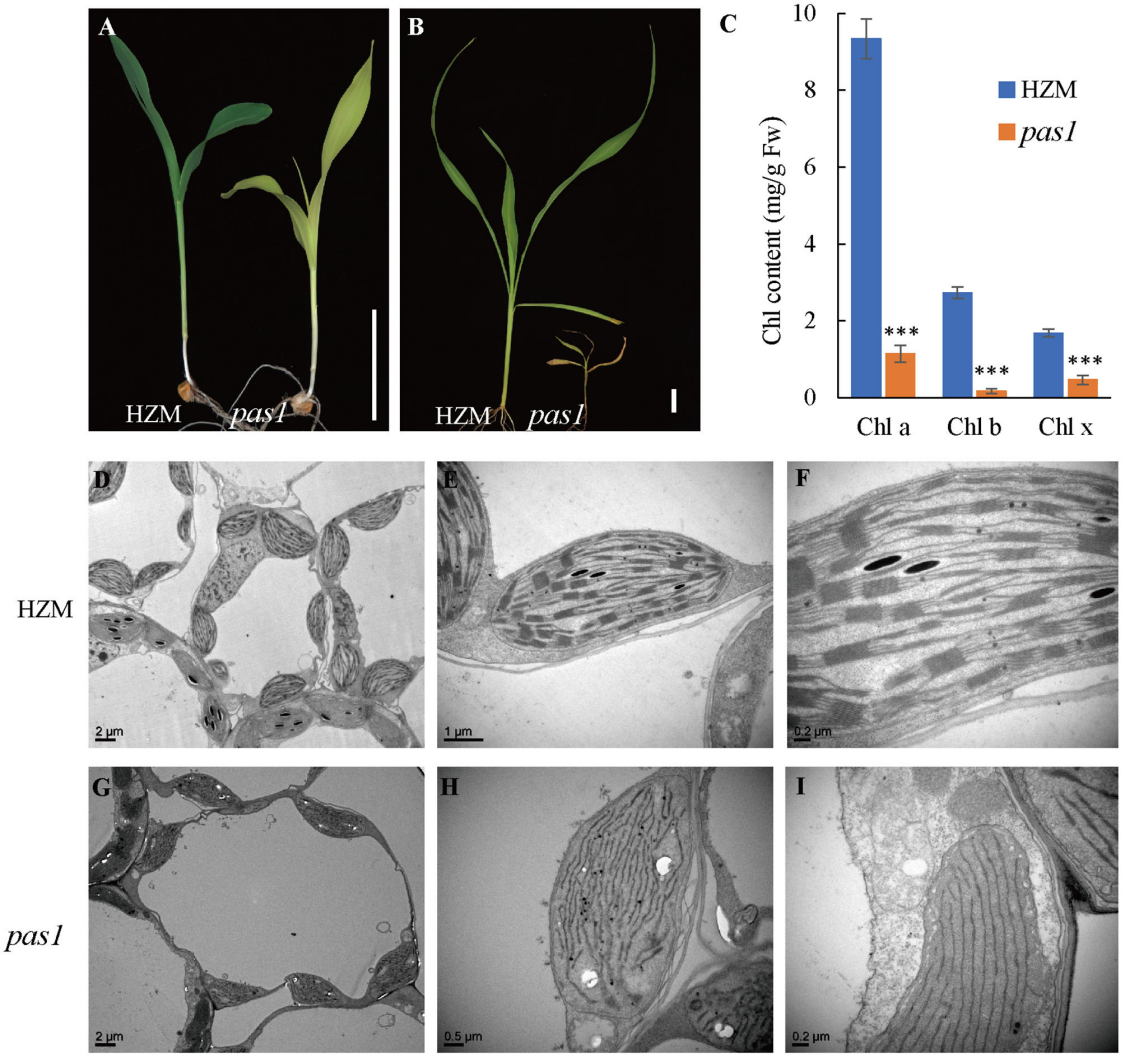
*pas1* is defective in plant growth and chloroplast development. **(A)**, Phenotype of wild-type and *pas1* mutant at the 3-leaf stage. Bar = 3 cm. **(B)**, Phenotype of the wild-type and *pas1* mutant at the 4-leaf stage. Bar = 3 cm. **(C)**, The chlorophyll contents of the wild-type and *pas1* mutant. **(D–I)**, Transmission electron microscopy for chloroplast structure of the wild-type and *pas1*. **(D–F)**, HZM; **(G–I)**, *pas1*.

To better understand the mechanisms beyond chloroplast development and photosynthesis of maize, we sought to screen new chlorosis mutants from a maize ethyl methane sulfonate (EMS) mutant library and identified a series of pale or light green leaf mutants. Among them, *pas1* displayed a severe chlorosis phenotype. Upon emergence from the germinating seeds, the *pas1* plants displayed light-yellow leaves and the growth was greatly retarded in comparison to the wild type seedlings (Figures 1A,B). The mutants barely survived beyond the four-leaf stage and only could be kept through segregation from heterozygous plants, indicating its defectiveness in photoautotrophic growth. Consistently, the chlorophyll contents were drastically reduced in the mutant (Figure 1C).

The failure of photoautotrophic growth implies the malfunction in the chloroplasts of the *pas1* mutant. Chloroplast structure of the wild type and mutant mesophyll cells were examined with transmission electron microscopy (TEM). As shown in Figures 1D–F, the chloroplasts of the wild-type plant contained well-organized lamellar and stacked thylakoid structures. In contrast, the thylakoid stacks were almost missing in *pas1* chloroplasts (Figures 1G–I).

### *ZmFdC2* Was Mutated in the *pas1* Mutant

**Figure 2:**
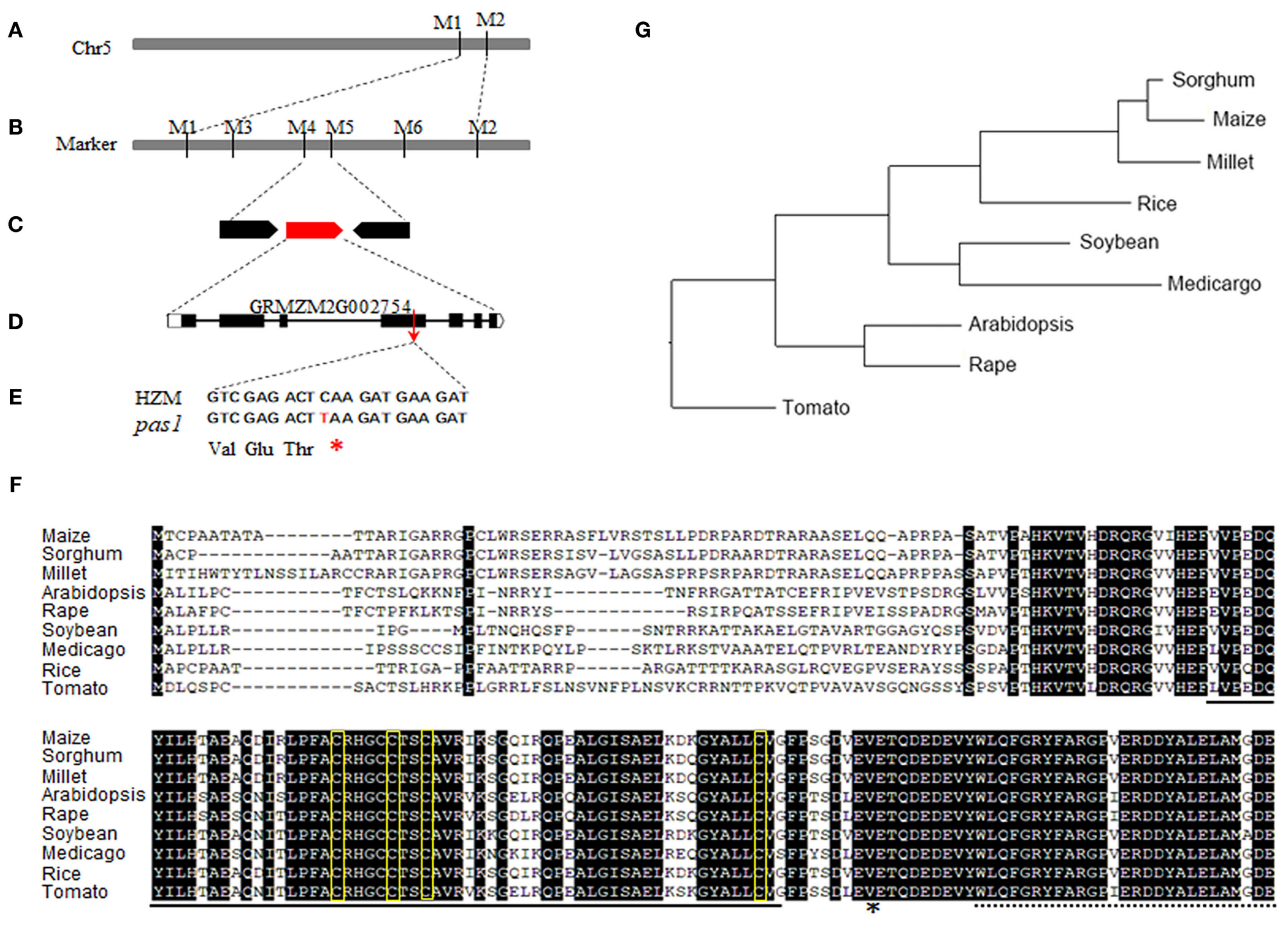
Map based cloning of *ZmFdC2* gene and the alignment of FdC2 proteins from various species. **(A)**, *ZmFdC2* gene was roughly mapped between marker M1 and M2. **(B)**, The *ZmFdC2* gene was further localized to a 104 kb region between M4 and M5. **(C)**, Three candidate genes were annotated in the mapped region. **(D)**, The diagram representation of the *ZmFdC2* gene structure. **(E)**, The C to T mutation in *ZmFdC2* gene led to an extra stop codon. **(F)**, Sequence alignment of FdC2 proteins from various species. The solid and dashed lines under the letters indicate the 2Fer-2S cluster and the C-terminus extensions, respectively. The amino acids in yellow rectangles denote the conserved –CxxxxCxxCxn–C– motif, and the asterisk indicates the mutated position in *ZmFdC2*. **(G)**, Phylogenetic tree of all FdC2 homologs from selected species.

To identify the causative gene of the *pas1* phenotype, we crossed the *pas1* heterozygous plants with Q319 inbred line. All F1 plants showed wild type phenotypes, and half of the F1 plants could segregate the *pas1* phenotype in the F2 generation. Nearly 25 percent of the F2 plants from the segregating lines displayed *pas1*-like phenotype (Supplementary Table 2), indicating that the mutant phenotype is controlled by a single recessive gene. Using the map based cloning approach and 910 F2 individual plants, we localized the gene locus to a 104-kilobase region on chromosome 5 (Figures 2A,B). To ensure that the region contains the causative gene, we generated a separate genetic population (*pas1* × Mo17) and mapped the locus to the same region. Gene annotation revealed 3 putative open reading frames in this region (Figure 2C). To identify the mutation, we amplified all the exons of each gene from the wild type (HZM) and the mutant plants, and applied Sanger sequencing. Sequence alignment identified only one gene (*Zm00001d013534*) with a point mutation (C475T) in the coding region (Figure 2D). The nucleotide substitution introduced an extra stop codon and resulted in a pre-mature protein, suggesting that this gene should be responsible for the *pas1* defective phenotype (Figure 2E).

*Zm00001d013534* encodes a peptide with 192 amino acids. Sequence blast showed that the protein belongs to the Fd family with the highest similarities to the FdC2 of rice and Arabidopsis, Thus, we named this gene *ZmFdC2* hereafter. We obtained FdC2 sequences from various plant species by homology search in Phytozome (https://phytozome.jgi.doe.gov/pz/portal.html). All FdC2 proteins contain the conserved 2Fe-2S cluster and the C-terminus extension (Figure 2F). Phylogenetic analysis showed that three C4 plants have higher similarities and belong to the same subclade (Figure 2G).

### ZmFdC2 Localizes on Chloroplast and Affects Photosynthetic Electron Transmitting

**Figure 3:**
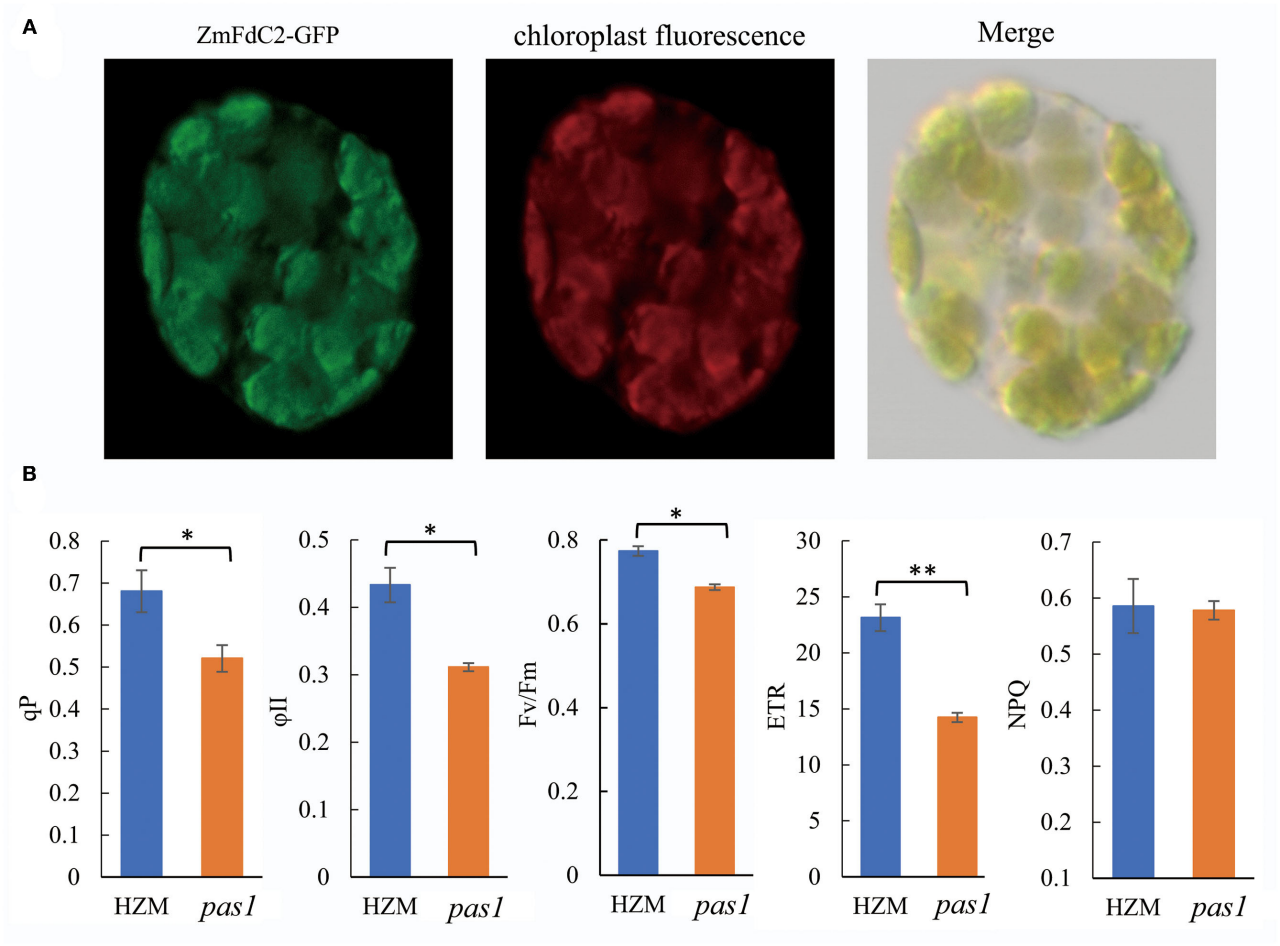
Chloroplast localization of ZmFdC2 and photosynthetic electron transport parameters of HZM and *pas1* mutant. **(A)**. Subcellular localization of ZmFdC2-GFP. *ZmFdC2-GFP* plasmid was transformed into maize protoplast, and fluorescence was detected with confocal microscopy. Left, ZmFdC2-GFP; middle, chloroplast autofluorescence; right, overlay of GFP and chloroplast signals. **(B)**, Photosynthetic electron transport parameters of HZM and *pas1* mutant. Chlorophyll fluorescence parameters for PSII capacity (ϕII), the photosynthetic electron transfer rate (ETR), photochemical quenching (qP), and non-photochemical quenching (NPQ) were determined. Values are mean ± S.D. of at least six independent measurements. Significances were determined with Student's *t-test*. “^*^” indicates *p* < 0.05 and “^**^” indicates *p* < 0.01.

To investigate the subcellular localization of ZmFdC2, we fused the GFP tag in frame with the C terminal of the ZmFdC2, and transiently expressed the plasmid in maize protoplast. Fluorescence signal under confocal microscopy showed that ZmFdC2-GFP fluorescence perfectly overlapped with chloroplast autofluorescence signals (Figure 3A). No fluorescence was detected on other subcellular structures, demonstrating that ZmFdC2 is a chloroplast specific protein.

Since ferredoxin proteins function as electron carriers in plant photosynthesis, we sought to determine whether *ZmFdC2* mutation could lead to alterations in PET. Chlorophyll fluorescence was measured to calculate the PET parameters in the wild-type plant and *pas1* mutant. As shown in Figure 3B, qP, ϕII, and Fv/Fm all decreased in the mutant, suggesting reductions in effective and maximum quantum yields of PSII. Furthermore, the electron transfer rate of PSII (ETR), was also reduced in the *pas1* mutant. In contrast, non-photochemical quenching (NPQ) showed little to no change compared to the wild-type plant.

### ZmFdC2 Interacts With Other Proteins in PET

**Figure 4:**
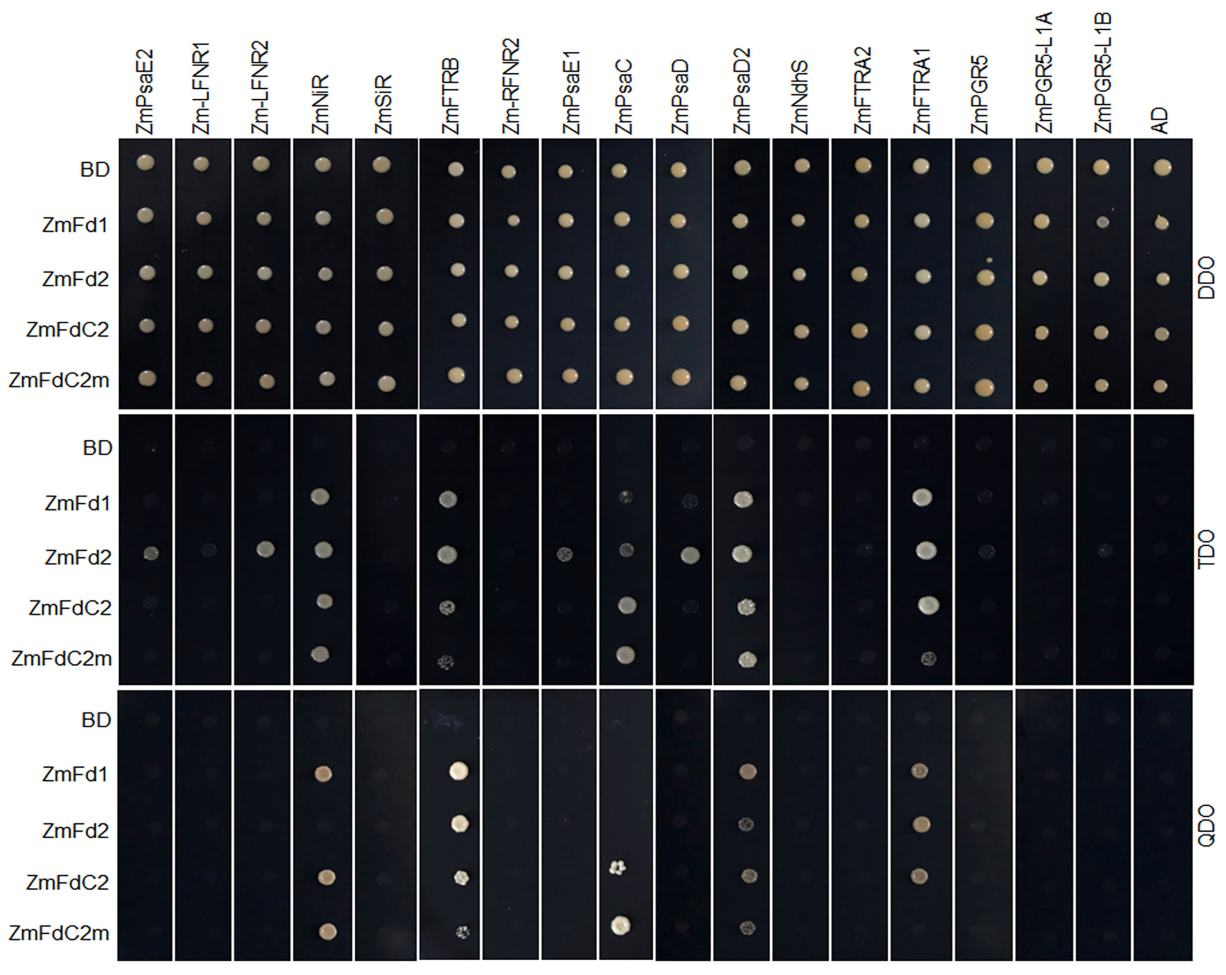
ZmFdC2 interacted with PET proteins of maize. *ZmFdI, ZmFdII, ZmFdC2* and *ZmFdC2m* were constructed into yeast two-hybrid bait plasmid (BD), and other maize PET genes were constructed into prey plasmid (AD). Yeast transformants were selected on DDO (SD/-Leu/-Trp) agar medium and the protein interactions were tested on TDO (SD/-Leu/-Trp/-His) and QDO (SD/-Leu/-Trp/-His/-Ade) agar medium.

As electron carriers in PET, ferredoxins accept electrons from PSI and subsequently transfer them to various acceptors. To test whether ZmFdC2 could interact with other PET components of maize, we cloned the 17 genes possibly involved in maize PET based on sequence similarities with the published Arabidopsis homologs and performed yeast two-hybrid experiments. ZmFdC2 was shown to interact with some PSI components such as ZmPsaD2 and ZmPsaC (Figure 4); it also interacted with some downstream acceptors including ZmNiR, ZmFTRA1 and ZmFTRB (Figure 4). The interactive partners were partially overlapped with those of ZmFd I and ZmFd II, the major electron carriers of maize PET. It is worthy to note that the mutation of *ZmFdC2* in this study altered its interactions with some partners (Figure 4).

### *ZmFdC2* Is Expressed in Photosynthetic Tissues and Induced by Light

**Figure 5:**
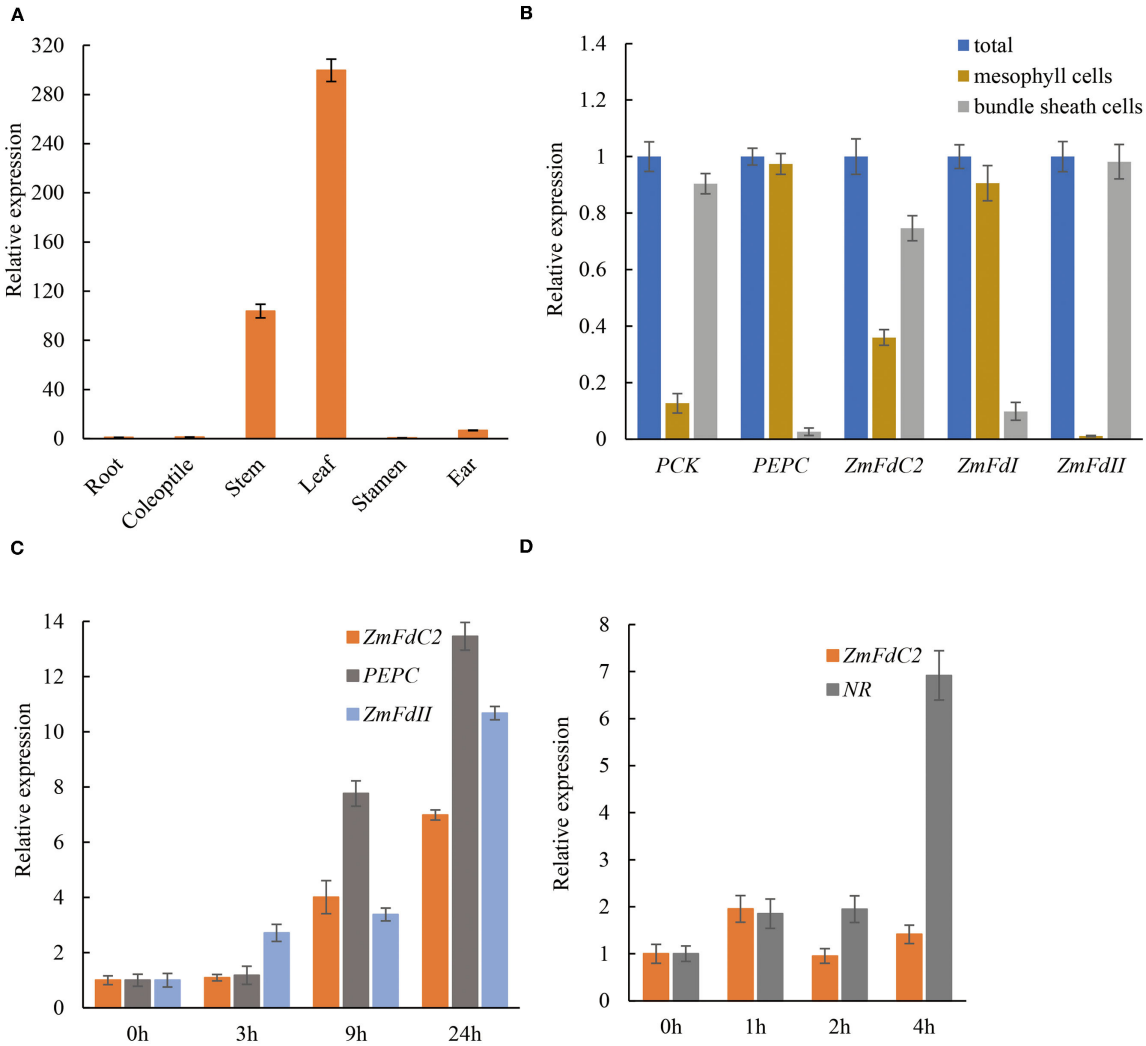
Tissue specificity and light induction of *ZmFdC2* expression. **(A)**, Real-time PCR of *ZmFdC2* in different maize tissues. All expression values in different tissues were normalized to that of root. **(B)**, Expression of different genes in mesophyll cells and bundle sheath cells of maize leaves. The expression of each gene in two cell types was normalized to its expression in the total RNA. *PEPC* and *ZmFdI* served as positive control for mesophylls; *PCK* and *ZmFdII* served as positive control for bundle sheath cells. **(C)**, Real-time PCR of *ZmFdC2* before and after light treatment. One-week-old seedlings grown in the dark were transferred to light and the youngest leaves were collected at different time points for RNA analyses. All expression values were normalized to that of 0 h of light treatment. **(D)**, Real-time PCR of *ZmFdC2* before and after nitrate addition. Two-week-old seedlings grown under low nitrate concentration (1.6 mM KNO_3_) were treated with 16 mM KNO_3_, and the youngest leaves were harvested at different time points for RNA analyses. All expression values were normalized to 0 h of nitrate treatment. All experiments were conducted for three biological replicates and results were presented as mean ± SD. Maize *Actin* gene served as internal control in all experiments except for **(B)**.

According to the expression specificity and entangled metabolism pathways, plant Fd genes could be roughly divided into photosynthetic and non-photosynthetic groups. To assess the role of *ZmFdC2*, we first examined RNA abundance in different tissues. As shown in Figure 5A, *ZmFdC2* was predominantly expressed in leaves and stems but barely detected in roots, indicating its specific function in photosynthetic pathways. As a C4 plant, maize accomplishes photosynthesis and CO_2_ assimilation in two different leaf tissues, mesophyll cells and bundle sheath cells. Different maize Fds were found to be specifically expressed in either of these tissues and participate in different steps of maize photosynthesis and PET. To better understand the function of ZmFdC2, we separated the mesophyll cells and bundle sheath cells from leaves of wild-type plants for RNA quantification. The two types of cells were well-separated based on the microcopy observation (Supplementary Figure 1). *PHOSPHOENOLPYRUVATE CARBOXYLASE* (*PEPC*) and *ZmFdI* were reported to be predominantly expressed in mesophyll cells, while *PHOSPHOENOLPYRUVATE CARBOXYKINASE* (*PCK*) and *ZmFdII* were found to be specifically expressed in bundle sheath cells. As shown in Figure 5B, the expression preferences of these genes are well-consistent with the previous studies, indicating that the separation of the two cell types is satisfactory. *FdC2* was expressed in both mesophyll and bundle sheath cells, implying its roles in C3 and C4 photosynthetic pathways.

A distinct feature of the photosynthetic Fd gene is the induced expression by light. To test whether *ZmFdC2* expression is light inducible, HZM seedlings were grown in the dark for a week, and then exposed to light for several hours. RNA abundances were quantified with real time PCR. Consistent with previous reports, maize *Fd II* and *PEPC* were significantly upregulated by light. *ZmFdC2* expression was also dramatically increased after a 9-h light treatment (Figure 5C). In addition, we also tested whether *ZmFdC2* expression could be altered by the addition of nitrate. As a positive control, the nitrate reductase gene (*NR*) was dramatically upregulated by nitrate, however, *ZmFdC2* expression remained unchanged after nitrate treatment (Figure 5D).

Taking these together, we identified *ZmFdC2* as a novel ferredoxin gene involved in maize photosynthetic electron transport and vital for maize photosynthesis and development.

### *ZmFdC2* Mutation Disturbed the Gene Expression of Photosynthesis and Other Metabolic Pathways

**Figure 6:**
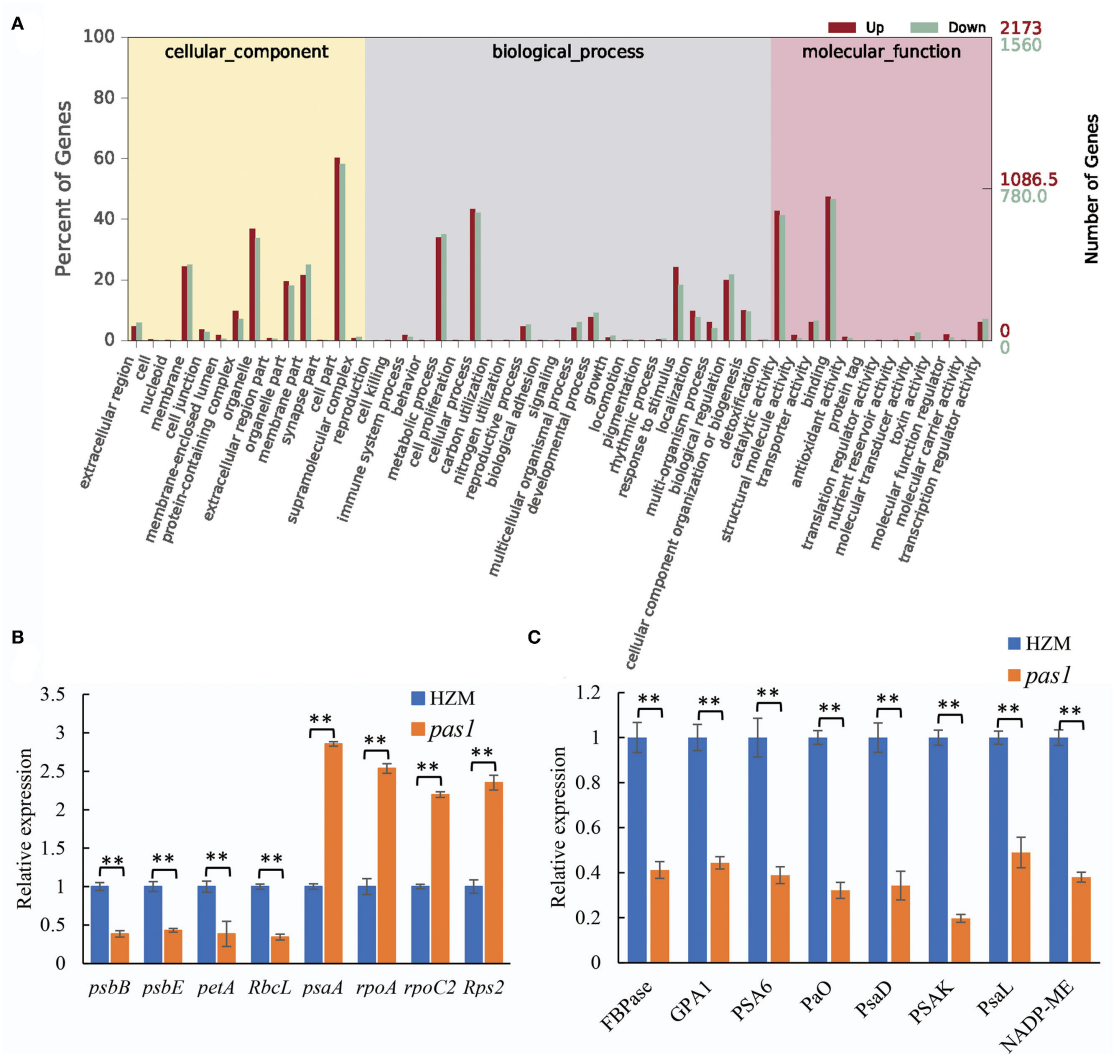
Differentially expressed genes in *pas1* mutant. The youngest leaves of seedlings at three-leaf stage were collected for the analyses. **(A)**, GO analysis of the DEGs from RNA-seq data between the wild-type and *pas1* mutant. RNA-seqs were performed for three biological replicates, and DEGs were defined as fold change > 2 and *p* < 0.05. **(B,C)**, expression validation of some DEGs from RNA-seq data. The selected DEGs included chloroplast-coding **(B)** and nuclear-coding **(C)** genes. The expression of each gene was normalized to HZM and maize *Actin* gene served as internal control. “^**^” indicates *p* < 0.01.

To better understand the mechanisms by which ZmFdC2 affects photosynthesis and plant growth, we performed mRNA sequencing on the wild-type and *pas1* mutant seedlings. Compared to the wild-type, 9,420 genes were up-regulated and 1952 were down-regulated in *pas1*, respectively. The differentially expressed genes (DEGs) were involved in diverse biological and metabolic pathways (Figure 6A). Although photosynthesis occurs in chloroplasts, the genes participating in this process are encoded by both nuclear and chloroplast genomes. RNA-seq data clearly showed that the expression of many photosynthesis-related genes encoded by both organelles were changed in the *pas1* mutant. To confirm the effects of *ZmFdC2* mutation on the expression of these genes, we collected the youngest leaves from the 7-day-old seedlings of wild-type and *pas1* plants and compared the RNA levels on selected genes with real-time PCR. 8 nuclear-coding and 8 chloroplast-coding genes were subjected to RNA quantification. Consistent with the RNA-seq result, the expressions of all tested genes were significantly altered in the mutant (Figures 6B,C).

### *ZmFdC2* Gene Is Associated With Active Histone Modification Marks

**Figure 7:**
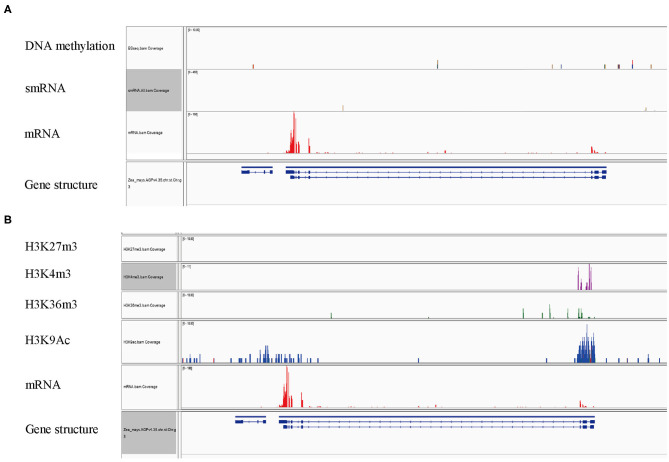
Epigenetic modifications associated with *ZmFdC2* locus. **(A)**, Reads of DNA methylation, mRNA and small RNA on *ZmFdC2* locus. **(B)**, Enrichments of four histone modifications on *ZmFdC2* locus. RNA-seq and all epigenetic data were generated from the same samples and downloaded from the publication (Wang et al., 2009).

The expression of many plant genes is under the control of epigenetic modifications. A landscape of epigenetic modifications on the maize genome has been generated and a vast number of genes were revealed to be differentially regulated by epigenetic modifications. To investigate whether *ZmFdC2* is associated with specific types of histone modification marks, we took advantage of published RNA-seq and epigenetic data on maize shoot tissues for the analysis (Wang et al., 2009). The *ZmFdC2* gene contains a 16 kb intron with features of an intact LTR transposon (data not shown). Transposon genes usually undergo DNA methylation for transcriptional silencing. This prompted us to look at the DNA methylation and heterochromatic siRNAs (hc-siRNA) around the gene. However, neither DNA methylation nor 24nt hc-siRNAs were abundantly detected around the locus, suggesting that *ZmFdC2* is not repressed by RNA dependent DNA methylation (RdDM) pathways (Figure 7A). Then we turned to check the enrichment of histone modification around the gene. H3K27me3, a repressive epigenetic mark, was absent at this locus, while three active epigenetic marks, H3K4m3e, H3K36me3, and H3K9Ac were enriched at the transcription start site (TSS) (Figure 7B). These results implied that the expression of *ZmFdC2* might be positively regulated by active epigenetic modifications.

## Discussion

Ferredoxin proteins play central roles in PET, which are critical for the formation of reducing power and ATP energy for the assimilation of CO_2_ and other metabolites. FdCs are recently identified proteins which showed high similarities with Fds, except for the C-terminus extension. Physiological and genetic studies demonstrated that FdC genes are involved in PET in Arabidopsis and rice (Voss et al., 2011; Li et al., 2015; Zhao et al., 2015; Guan et al., 2018). Homology search and phylogenetic analyses predicted two maize FdC genes in maize genome named *ZmFdC1* and *ZmFdC2*, respectively. However, the function of these genes had not been reported. Here, we identified the *ZmFdC2* gene through forward genetics study. The gene is expressed in both mesophyll and bundle sheath cells of maize, and its mutation resulted in impaired PET and caused seedling death, demonstrating its critical role in maize development.

The mutation in *ZmFdC2* might interrupt its interactions with coenzymes. Wild-type *ZmFdC2* encodes a peptide composed of 192 amino acids. The mutation introduced an extra stop codon and resulted in a pre-mature protein of 158 amino acids. Domain annotation revealed that the mutant protein contains the full 2F2-2S domain, and the N-terminal sequence harbors the plastid transit peptide (Figure 2F). That is, the mutant version of ZmFdC2 contains enough information for chloroplast localization and electron transfer. Considering the reduced PET rate and the severe developmental defect of the mutant, it is possible that the lost 34 amino acids at the C-terminal is vital to the normal function of ZmFdC2. Several possibilities might account for these defects. First, the truncation of C-terminus amino acids affected the expression of *ZmFdC2*. To rule out this possibility, we compared the RNA accumulation of *ZmFdC2* in the wild-type and the mutant but did not find significant differences (Supplementary Figure 2). Second, the missing C-terminal amino acids might be important for electron accepting or transferring. In agreement with this, the simulation of protein structure on ZmFdC2 protein indicated that the mutation obviously altered the confirmation in the C terminus region (Supplementary Figure 3). The yeast two-hybrid results showed that the mutation indeed led to alterations of protein interactions between ZmFdC2 and PSI electron components or downstream acceptors (Figure 4). The third possibility is that the C-terminus extension is important for specialized functions of FdC. FdC proteins share high similarity with Fds except for the C-terminus extensions. Evidence from physiology and biochemistry in Arabidopsis and rice indicated that FdCs were functional electron transmitters (Voss et al., 2011; Guan et al., 2018). Both Fds and FdCs are conserved across the plant kingdom, but the function of the extension region in the FdC proteins is largely unknown. Lack of mutants exactly missing the C-terminus extension hindered the precise evaluation of the C-terminus function. In this study, ZmFdC2 lost the entire C-terminus region plus nine amino acids between the 2Fe-2S cluster and the C-terminus region. Although these nine amino acids may interrupt interactions with electron donors or acceptors, it is still possible that the C-terminal extension is critical for its specialized activity.

ZmFdC2 is a photosynthetic type of ferredoxin protein. Several lines of evidence support the fact that ZmFdC2 participates in photosynthetic electron transport. First, *ZmFdC2* is exclusively expressed in photosynthetic tissues including leaves and stems. In maize, *ZmFdI* and *II* are expressed in leaves and transfer electrons to FNR for NADPH production and CO_2_ assimilation. On the other hand, *ZmFdIII, IV*, and *VI* are expressed in roots and transfer electrons to nitrite or sulfite reductase for nitrate or sulfate assimilation (Matsumura et al., 1997; Sakakibara, 2003). *ZmFdC2* expressions are specifically detected in leaves and stems, indicating its roles in photosynthesis. Second, ZmFdC2 is localized in the chloroplast where photosynthesis takes place, and the gene mutation led to the thinning or disappearance of thylakoid stacks in the chloroplast. This defect resulted in the malfunction of the chloroplast, which could be proved by the pale leaves and seedling death of the mutant. Third, the expression of *ZmFdC2* is strongly induced by light treatment. Photosynthetic and non-photosynthetic ferredoxins respond distinctively to different environmental signals. As a non-photosynthetic ferredoxin gene, maize *ZmFd VI* expression could be quickly induced by nitrate, but not by light treatment (Matsumura et al., 1997). The expression of *ZmFdC2* was not responsive for nitrate addition; Instead, its expression was strongly stimulated by light, which is very similar to the responses of *ZmFd I* and *ZmFd II*, the two photosynthetic ferredoxin genes in maize.

ZmFdC2 is indispensable for maize growth and development. So far, the function of the C-terminus extension of FdC is poorly understood. Arabidopsis FdC1 and rice FdC2 were demonstrated to be involved in PET. However, *AtFdC1* mutation did not show obvious alteration in growth compared with the wild-type plants (Guan et al., 2018); *OsFdC2* mutant displayed yellow leaves and delayed flowering but was still viable and fertile, indicating their limited roles or functional redundancies in plant development (Li et al., 2015; Zhao et al., 2015). In contrast, mutation of *ZmFdC2* resulted in seedling death of the maize plant, implying its more important or specialized role in maize development. The expression profile revealed that *ZmFdC2* is expressed in both mesophyll and bundle sheath tissues, suggesting spatial overlapping of the expression profiles of *ZmFdC2* with *ZmFdI* and *II*, the two Fd genes specifically expressed in mesophyll and bundle sheath cells, respectively (Sakakibara, 2003). Surprisingly, the *ZmFdC2* mutant was lethal at the seedling stage, indicating the failure of the functional complementation by Zm*Fd I* or *II*. After accepting electrons from PSI, Fds will distribute them to different co-enzymes for downstream metabolic activities. The well-known co-enzyme in photosynthetic electron transmitting is FNR, which uses flavin adenine dinucleotide (FAD) as a cofactor. Maize FdI has been demonstrated to donate the electrons to FNR for CO_2_ assimilation. The C-terminus extensions in FdCs may interfere with their interactions with downstream electron acceptors. For example, AtFdC1 is unfavorable to the binding with FNR. Instead, it receives electrons from PSI and transfers them to ferredoxin-thioredoxin reductase (FTR), NiR or SiR(Guan et al., 2018). Consistently, ZmFdC2 didn't interact with maize FNRs, instead, it interacted with ZmNiR, ZmFTRA1, ZmFTRB and PSI components ZmPsaD2 and ZmPsaC (Figure 4). In chloroplast, water is split by light energy at the lumenal oxygen evolving complex. The generated electrons are transmitted from PSII to PSI. In PSI, PsaC, PsaD and PsaE form the so-called “stromal ridge,” which contacts and transmits the electrons to Fds. Generally, PsaD guides Fds to the binding site of PSI, PsaE stabilizes the complex and PsaC mediates the transfer of electrons to Fd proteins (Hanke and Mulo, 2013). FTRs are involved in the regulation of various key enzymes such as fructose-1,6-bisphosphatase (FBPase) and NADP-dependent malate dehydrogenase (NADP-MDH) (Dai et al., 2000). The interaction of ZmFdC2 with the PSI components, FTRs and NiR implies its possible functions in the assimilation of carbon and nitrate of maize. As a C4 plant, maize accomplishes photosynthesis in two different tissues. In mesophyll chloroplast, CO_2_ is used to carboxylate PEP and form malate acid, which is defused into bundle sheath chloroplast to release CO_2_ for Calvin cycle. To sustain this process, the pyruvates are transported back to mesophyll cells for PEP regeneration (Schluter and Weber, 2020). This process requires more ATPs than C3 photosynthesis. CEF in bundle sheath cells of C4 plant is important to meet this requirement because when electrons are transferred back to PSI from Fds, a larger proton gradient could be generated and produce extra ATP (Yin and Struik, 2018). *ZmFdC2* is abundantly expressed in bundle sheath cells in addition to mesophyll cells (Figure 5B). Bundle sheath is thought to be important for CEF in C4 plants. AtPGR5 has been demonstrated to mediate the CEF in Arabidopsis, however, our results in yeast did not support the interaction of ZmFdC2 and ZmPGR5 or its homologs (Figure 4), suggesting that ZmFdC2 may not involve in CEF, although it is still possible that ZmFdC2 may interact with other unidentified CEF components.

## Accession Numbers and Gene IDs

NCBI gene ID: *ZmFdC2*, Zm00001d013534. NCBI accession numbers of FdC2 homologs: Tomato, XP_004245886.1; Arabidopsis, NP_174533.1; Rape, XP_009107976.1; Soybean, RZC11990.1; Medicago, XP_003608621.1; Rice, XP_015629147.1; Sorghum, XP_021306920.1; Maize, XP_008644599.1; Millet, RCV41454.1. Putative PET genes in maize: *ZmPsaC*: GRMZM2G096792; *ZmPsaD*, GRMZM2G013342; *ZmPsaE1*, GRMZM2G016622; *ZmPsaD2*, GRMZM2G024150; *ZmPsaE2*, GRMZM2G016066; *ZmLeafFNR1*, GRMZM2G168143; *ZmLeafFNR2*: GRMZM2G059083; *ZmNdhS*, AC234522.1; *ZmNiR*, GRMZM2G079381; *ZmSiR*, GRMZM2G090338; *ZmPGR5-Like1B*, GRMZM5G885392; *ZmPGR5-Like1A*, GRMZM5G896082; *ZmFTRB*, GRMZM2G122793; *ZmFTRA2*, GRMZM2G157458; *ZmFTRA1*, GRMZM2G139803; *ZmRootFNR1*, GRMZM2G015352; *ZmRootFNR2*, GRMZM2G058760; *ZmPGR5*, GRMZM2G017045.

The Bioproject number of the RNA seq data in this paper is PRJNA689125.

## Data Availability Statement

The datasets presented in this study can be found in online repositories. The names of the repository/repositories and accession number(s) can be found below: NCBI, PRJNA689125.

## Author Contributions

SL designed the experiments and wrote the paper. LG performed the genomic sequencing analyses. YC conducted gene cloning and other molecular experiments and participated the paper writing. DZh screened the mutant library and generated mapping population. YZ and QW performed the yeast two-hybrid experiments. LD and DZe performed chlorophyll fluorescence determination. XY assisted in cloning of *ZmFdC2* gene. All authors discussed and revised the manuscript.

## Conflict of Interest

The authors declare that the research was conducted in the absence of any commercial or financial relationships that could be construed as a potential conflict of interest. The reviewer YW declared a shared affiliation, with no collaboration, with several of the authors, SL and YZ, to the handling editor at the time of the review.

## Supplementary Material

Supplementary Figure 1 Separation of bundle sheath cells and mesophyll cells. The two types of cells were observed under light microscope. Round cells on the right are the mesophyll protoplast cells, and the tubelike structures on the left are the bundle sheath tissue.

Supplementary Figure 2 *ZmFdC2* expression in HZM and *pas1*. The youngest leaves of 7-day-old seedlings from HZM and *pas1* were subjected for RNA isolation. Gene expression was normalized to HZM. Maize *Actin* gene served as internal control. The quantifications were from three biological replicates.

Supplementary Figure 3 3-D structure simulation of ZmFdC2 and ZmFdC2m proteins. N and C indicated N- and C-terminus of the proteins.

Supplementary Table 1 Primers used in this study.

Supplementary Table 2 Segregation of F_2_ populations.
