# Supplementary figures and images for: ZmFdC2 Encoding a Ferredoxin Protein With C-Terminus Extension Is Indispensable for Maize Growth
Source: Front Plant Sci. 2021 Apr 23;12:646359. doi: 10.3389/fpls.2021.646359 (PMC8104031; doi:10.3389/fpls.2021.646359)

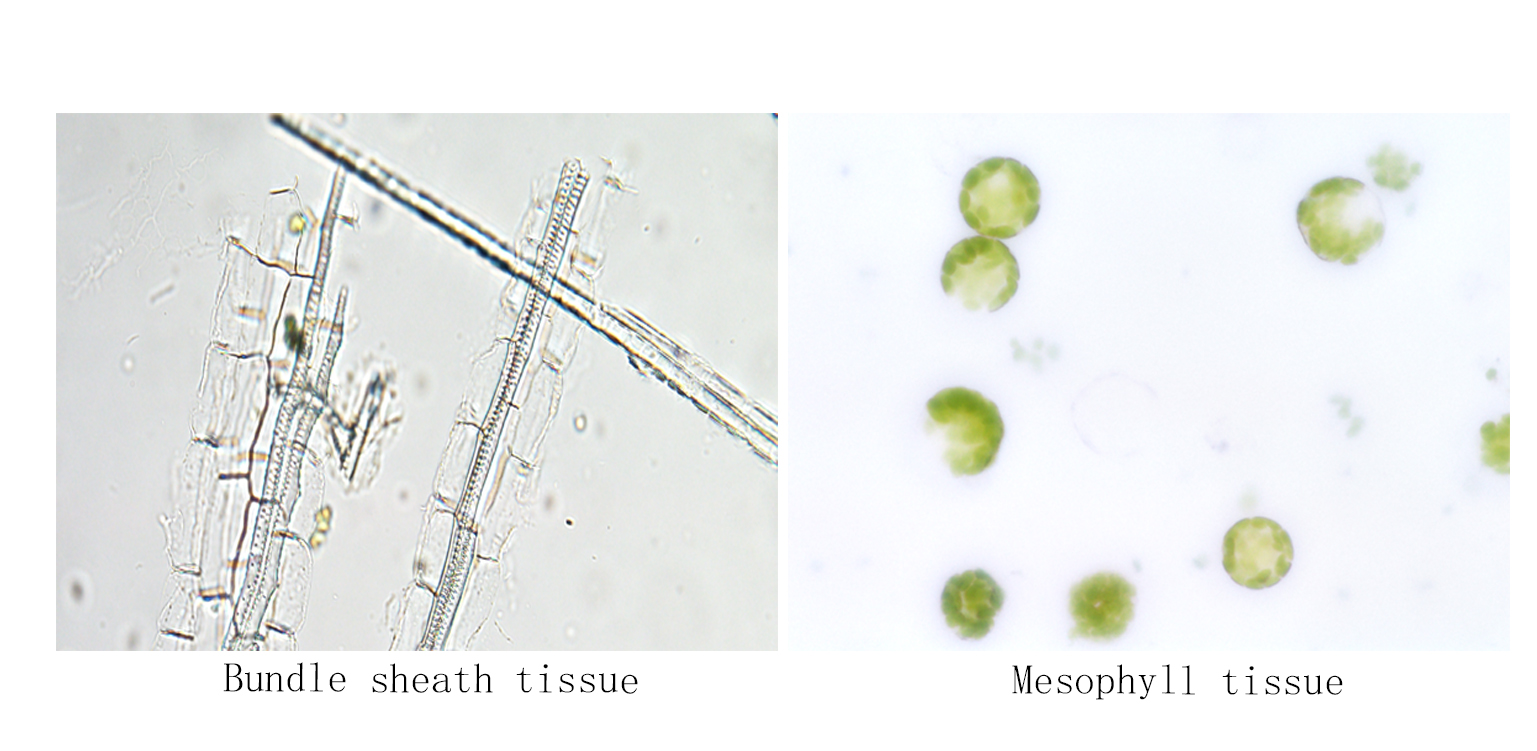

Supplement: Supplementary Figure 1 — Separation of bundle sheath cells and mesophyll cells. The two types of cells were observed under light microscope. Round cells on the right are the mesophyll protoplast cells, and the tubelike structures on the left are the bundle sheath tissue. [file Image_1.JPEG]

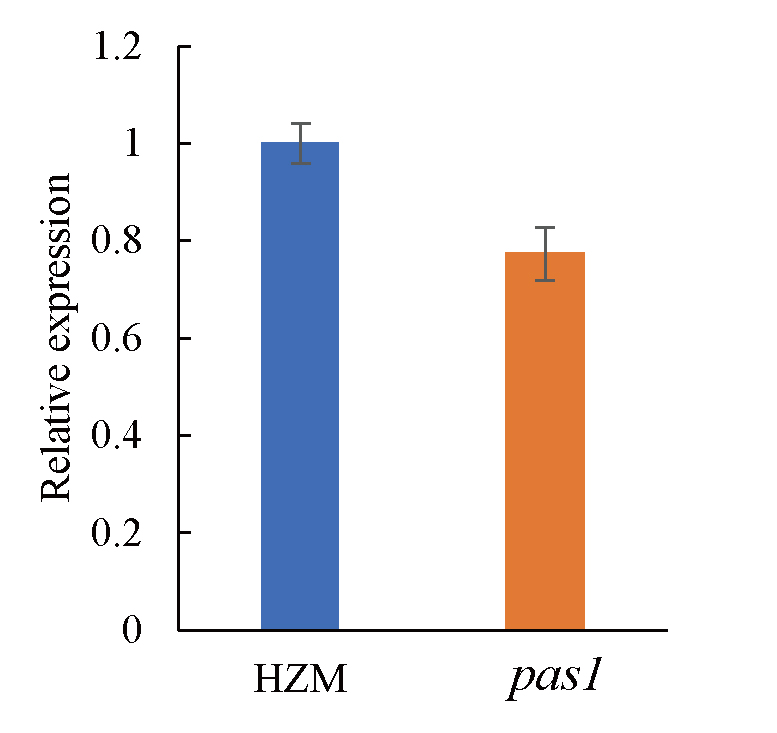

Supplement: Supplementary Figure 2 — ZmFdC2 expression in HZM and pas1. The youngest leaves of 7-day-old seedlings from HZM and pas1 were subjected for RNA isolation. Gene expression was normalized to HZM. Maize Actin gene served as internal control. The quantifications were from three biological replicates. [file Image_2.JPEG]

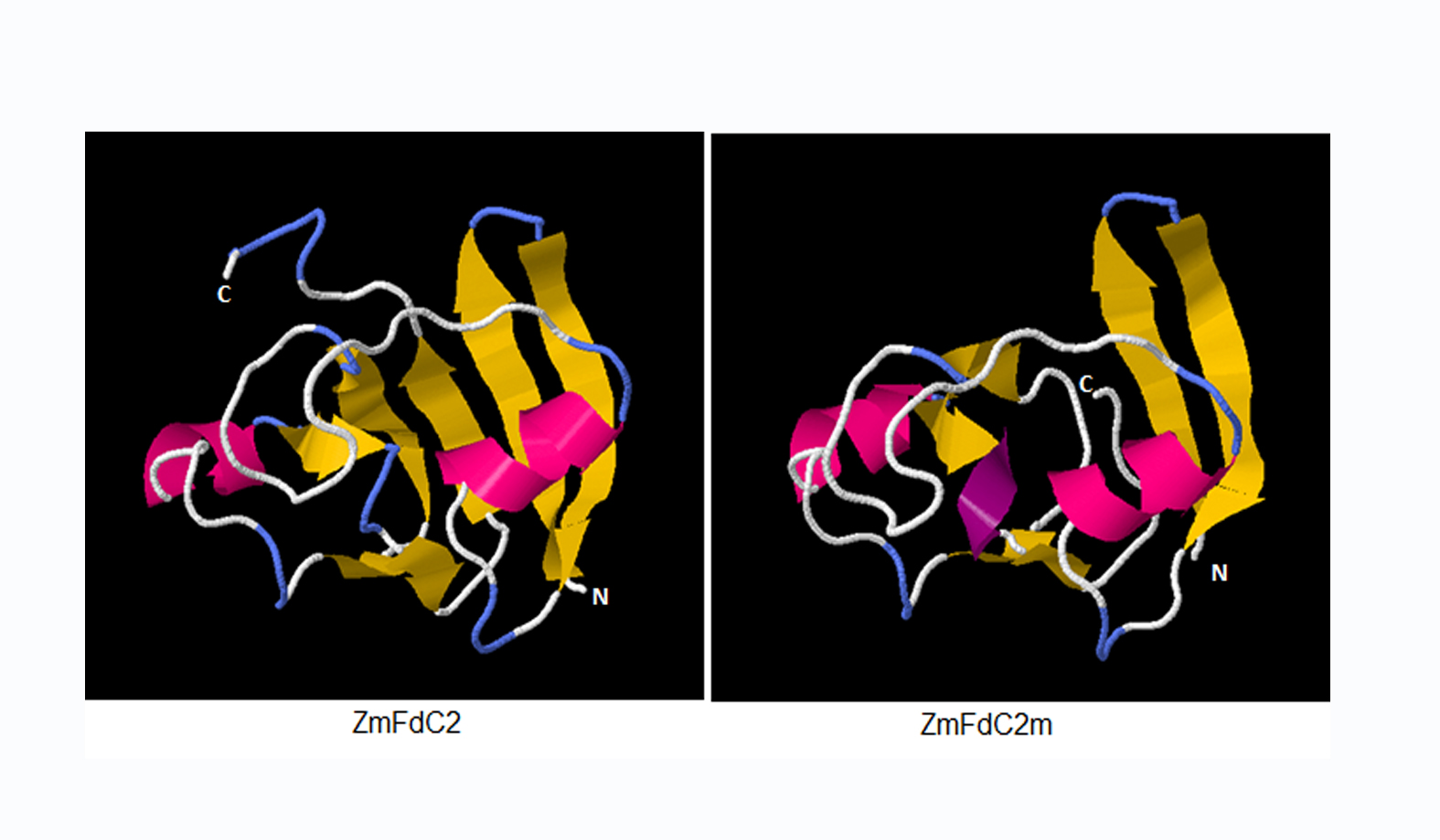

Supplement: Supplementary Figure 3 — 3-D structure simulation of ZmFdC2 and ZmFdC2m proteins. N and C indicated N- and C-terminus of the proteins. [file Image_3.JPEG]
