# Supplementary material for: ZmFdC2 Encoding a Ferredoxin Protein With C-Terminus Extension Is Indispensable for Maize Growth
Source: Front Plant Sci. 2021 Apr 23;12:646359. doi: 10.3389/fpls.2021.646359 (PMC8104031; doi:10.3389/fpls.2021.646359)
Supplement: Supplementary Table 1 — Primers used in this study. [file Table_1.DOCX]

Table S1 Primers used in this study

| **Gene_ID** | **Primer Name** | **Primer Sequence (5’-3’)** | **Purpose** |
| --- | --- | --- | --- |
|  | M1-F | AAGATGGTCAGCTAGAGCG | Map based cloning |
|  | M1-R | TTGGTAGGATTTAAGCAGACA | Map based cloning |
|  | M2-F | TAATAGTAATCAGAGGAGCA | Map based cloning |
|  | M2-R | GCAATCAATCAATAAGGCATT | Map based cloning |
|  | M3-F | GGGTCAGGTCACTTTTACTCG | Map based cloning |
|  | M3-R | ACGGAGGACAAAAAAAAATACA | Map based cloning |
|  | M4-F | AGGCTAGAAGAGCGGCAGCCC | Map based cloning |
|  | M4-R | AGGTGCGGTGCGGTTGGTTCTC | Map based cloning |
|  | M5-F | GTTATCATGGCTGGTGCGTCT | Map based cloning |
|  | M5-R | AGAGAGCTTCAGATAAACGCGA | Map based cloning |
|  | M6-F | AGATTATGCTGCCAACATAAT | Map based cloning |
|  | M6-R | CACTGCCCATTTCTGTTTTAT | Map based cloning |
|  | DORFdC2-F | GgggacaagtttgtacaaaaaagcaggcttaTCAATC  AGGTTTCAGGCCGAC | FdC2-GFP construction |
|  | DORFdC2-R | GgggaccactttgtacaagaaagctgggtaTTAGATG  GCAAGGTCTCTTAGGC | FdC2-GFP construction |
| Zm00001d046170 | PEPC-F | ATATCGGCATCAATGAGCGC | Real time PCR |
|  | PEPC-R | GGCCAGCAAGCATACATCTC | Real time PCR |
| GRMZM2G122337 | FdI-F | TTCTTCTTCTCGCCGTCCTC | Real time PCR |
|  | FdI-R | TGGTCCAGGATGTACACGTC | Real time PCR |
| GRMZM2G048313 | FdII-F | ACGACGTCTACATCCTGGAC | Real time PCR |
|  | FdII-R | CTGGTTGTCGTTGAGGAAGC | Real time PCR |
| AB018744 | PCK-F | GTCCACGAGCATAGACCTCA | Real time PCR |
|  | PCK-R | TGAGAGGCCAAAGAAGAGGG | Real time PCR |
| GRMZM2G126010 | ZmActin-F | GATGATGCGCCAAGAGCTG | Real time PCR |
|  | ZmActin-R | GCCTCATCACCTACGTAGGCAT | Real time PCR |
| GRMZM2G002754 | FdC2-F | ATCTGGCGATGTTGAAGTCGAGAC | Real time PCR |
|  | FdC2-R | CAAATTTGAGATGGTATTCAAAT | Real time PCR |
| GRMZM2G568636 | NR-F | AAGGTCTACTTCAAGAACGAGC | Real time PCR |
|  | NR-R | TACTTCATCTTCTCCAAGTTGGG | Real time PCR |
| Zm00001d000282 | psbB-F | TTGATCCTTTCGTTCCGGGA | Real time PCR |
|  | psbB-R | CGGACGGACACTTAGATGGA | Real time PCR |
| GRMZM5G801958 | psbE-F | TCTGGAAGCACGGGAGAAC | Real time PCR |
|  | psbE-R | ACCAACCCGCAATGAATAGG | Real time PCR |
| GRMZM2G085236 | petA-F | GGTTGAATGTGGGTGCTGTT | Real time PCR |
|  | petA-R | GGGAACAGGGCCTATCACAA | Real time PCR |
| GRMZM2G087628 | RbcL\-F | GTATTCGCTGAGGTGCTTGG | Real time PCR |
|  | RbcL-R | TCCTGTCCACGAGCTCAAAT | Real time PCR |
| Zm00001d000272 | psaA-F | CGGCCACCAGAGAATTCTTG | Real time PCR |
|  | psaA-R | TCCACCTTGTCCACTTCCTC | Real time PCR |
| GRMZM2G570791 | rpoA-F | AGGTCAAGCGGATACTGTCG | Real time PCR |
|  | rpoA-R | AATTTGGCGCGTGTGATACA | Real time PCR |
| GRMZM5G892247 | rpoC2-F | GCCTGACATTGTGATCGTCC | Real time PCR |
|  | rpoC2-R | GTCATAGTTGGCAGGAATCG | Real time PCR |
| GRMZM2G032602 | Rps2-F | AGTCTGTCGGGGTTCTCTTG | Real time PCR |
|  | Rps2-R | CCTTCCTTCCTCGGTCAGTT | Real time PCR |
| GRMZM2G306732 | FBPase-F | CATGTGAAGGACACCACGAC | Real time PCR |
|  | FBPase-R | TCCAGTGTAGCGCAATGTGT | Real time PCR |
| GRMZM2G162529 | GPA1-F | TGCTCTTACCCTGGCATCAA | Real time PCR |
|  | GPA1-R | GTCTGTCGAATTGCCCATCC | Real time PCR |
| GRMZM2G012397 | PSA6-F | CGTCGCCGTTGTTAAGATCA | Real time PCR |
|  | PSA6-R | CGCACACACGTTACAAAGTC | Real time PCR |
| Zm00001d011819 | PaO-F | TTCTCAGGTGCCGATAGCTT | Real time PCR |
|  | PaO-R | CTGGATCCTACCCTCGTTCA | Real time PCR |
| GRMZM2G013342 | PsaD-F | GGTGGAGGAGTTCTACGTCATC | Real time PCR |
|  | PsaD -R | AGACGCGGTAGAACTGGTAGGT | Real time PCR |
| GRMZM2G012397 | PSAK-F | CGTCGCCGTTGTTAAGATCA | Real time PCR |
|  | PSAK-R | CGCACACACGTTACAAAGTC | Real time PCR |
| GRMZM2G094224 | PsaL-F | AAGTTCACCGGAGGGTTCTT | Real time PCR |
|  | PsaL-R | ATCGGAGGCGGAGCTATATT | Real time PCR |
| GRMZM2G085019 | NADP-me-F | AGCGAATCTTGGGACTTGGA | Real time PCR |
|  | NADP-me-R | GCAAGCAAACTGATGGGTCA | Real time PCR |
| GRMZM2G016066 | ZmPsaE2-F | gccatggaggccagtgaattcATGGCGAGCACCAACATGG | Yeast two-hybrid |
|  | ZmPsaE2-R | cagctcgagctcgatggatccTCATTTCACCTCGAGGATCTCG | Yeast two-hybrid |
| GRMZM2G168143 | Zm-LFNR1-F | gccatggaggccagtgaattcATGGCCACCGTCATGGCC | Yeast two-hybrid |
|  | Zm-LFNR1-R | cagctcgagctcgatggatccTTAGTAGACCTCCACATTCCATTGA | Yeast two-hybrid |
| GRMZM2G059083 | Zm-LFNR2-F | gccatggaggccagtgaattcATGGCTGCCGTGACCGCG | Yeast two-hybrid |
|  | Zm-LFNR2-R | cagctcgagctcgatggatccTCAGTAGACTTCGACGTTCCATTG | Yeast two-hybrid |
| GRMZM2G079381 | ZmNiR-F | gccatggaggccagtgaattcATGGCCTCCTCAGCGTCC | Yeast two-hybrid |
|  | ZmNiR-R | cagctcgagctcgatggatccCTACTCCTCATCCTCCTCCCTCTC | Yeast two-hybrid |
| GRMZM2G090338 | ZmSiR-F | gccatggaggccagtgaattcATGTCGGGGGCGATCGGG | Yeast two-hybrid |
|  | ZmSiR-R | cagctcgagctcgatggatccTCATGCGGCTGATGGTGAC | Yeast two-hybrid |
| GRMZM2G122793 | ZmFTRB-F | gccatggaggccagtgaattcATGACATCCACCGTCACCACA | Yeast two-hybrid |
|  | ZmFTRB-R | cagctcgagctcgatggatccTCATAGAAAAAATAGCTGGAAGTGTAGTT | Yeast two-hybrid |
| GRMZM2G058760 | Zm-RFNR2-F | gccatggaggccagtgaattcATGGCGACCGCTGCTGCT | Yeast two-hybrid |
|  | Zm-RFNR2-R | cagctcgagctcgatggatccCTAGTAGACCTCGACGTGCCATT | Yeast two-hybrid |
| GRMZM2G016622 | ZmPsaE1-F | gccatggaggccagtgaattcATGGCGAGCACCAACATGG | Yeast two-hybrid |
|  | ZmPsaE1-R | cagctcgagctcgatggatccTCATTTCACCTCTGAGACCTC | Yeast two-hybrid |
| GRMZM2G096792 | ZmPsaC-F | gccatggaggccagtgaattcATGTCACATTCTGTAAAAATTTATGATACA | Yeast two-hybrid |
|  | ZmPsaC-R | cagctcgagctcgatggatccTCAATAAGATAGAGCCATGCTGCG | Yeast two-hybrid |
| GRMZM2G013342 | ZmPsaD-F | gccatggaggccagtgaattcATGGCCATGGCCACGCAA | Yeast two-hybrid |
|  | ZmPsaD-R | cagctcgagctcgatggatccTTAGTCGAAGGTGGCCTTGC | Yeast two-hybrid |
| GRMZM2G024150 | ZmPsaD2-F | gccatggaggccagtgaattcATGCTCGCGCCGCCCTCT | Yeast two-hybrid |
|  | ZmPsaD2-R | cagctcgagctcgatggatccTTAGTCGAAGGTGCTCTTGCCG | Yeast two-hybrid |
| AC234522.1 | ZmNdhS-F | gccatggaggccagtgaattcATGGCGCCGCCACCCACC | Yeast two-hybrid |
|  | ZmNdhS-R | cagctcgagctcgatggatccTCATGTCGCCGCCGCCGC | Yeast two-hybrid |
| GRMZM2G157458 | ZmFTRA2-F | gccatggaggccagtgaattcATGGCGACCGCCACCGCG | Yeast two-hybrid |
|  | ZmFTRA2-R | cagctcgagctcgatggatccTCACTCCCCATCGACGAACTC | Yeast two-hybrid |
| GRMZM2G139803 | ZmFTRA1-F | gccatggaggccagtgaattcATGGCGACCGCCACCGCG | Yeast two-hybrid |
|  | ZmFTRA1-R | cagctcgagctcgatggatccTCACTCTCCGTCGACGAACTCG | Yeast two-hybrid |
| GRMZM2G017045 | ZmPGR5-F | gccatggaggccagtgaattcATGGCGGCGGCTGTGTCC | Yeast two-hybrid |
|  | ZmPGR5-R | cagctcgagctcgatggatccTCAGGCAAGGAATCCGAGCTTCT | Yeast two-hybrid |
| GRMZM5G896082 | ZmPGR5-L1A-F | gccatggaggccagtgaattcATGGCGTCCGAGGTGTGG | Yeast two-hybrid |
|  | ZmPGR5-L1A-R | cagctcgagctcgatggatccTTATGCATTTGATGCTTCTGGAA | Yeast two-hybrid |
| GRMZM5G885392 | ZmPGR5-L1B-F | gccatggaggccagtgaattcATGGCCACCCAATATGCGC | Yeast two-hybrid |
|  | ZmPGR5-L1B -R | cagctcgagctcgatggatccTTATTTAGCCTCAGCCGGTTCT | Yeast two-hybrid |
| GRMZM2G122337 | ZmFd1-F | CATGCCATGGCAATGGCCACCGTCCTAGGCA | Yeast two-hybrid |
|  | ZmFd1-R | CGGGATCCTTATGCGCCGGTGAGCTCCT | Yeast two-hybrid |
| GRMZM2G048313 | ZmFd2-F | CATGCCATGGCAATGGCCGCCACCGCCCTGA | Yeast two-hybrid |
|  | ZmFd2-R | CGGGATCCTTATAGGAGGTCATCCTCCTTGTGC | Yeast two-hybrid |
| GRMZM2G002754 | ZmFdC2-F | CATGCCATGGCAATGACCTGCCCCGCCGCCA | Yeast two-hybrid |
|  | ZmFdC2-R | CGGGATCCTCATTCGTCTCCCATTGCAAGTTCTA | Yeast two-hybrid |
|  | ZmFdC2m-R | CGGGATCCTCATTCAACATCGCCAGATGGGAAACC | Yeast two-hybrid |
