# Supplementary material for: ZmFdC2 Encoding a Ferredoxin Protein With C-Terminus Extension Is Indispensable for Maize Growth
Source: Front Plant Sci. 2021 Apr 23;12:646359. doi: 10.3389/fpls.2021.646359 (PMC8104031; doi:10.3389/fpls.2021.646359)
Supplement: Supplementary Table 2 — Segregation of F2 populations. [file Table_2.DOCX]

Table S2 Segregation of F_2_ populations

| Cross | Q319^a^/*pas1* |
| --- | --- |
| Numbers of normal plants^b^ | 2620 |
| Numbers of pale plants | 910 |
| Total numbers | 3530 |
| χ2 | 1.143 |
| P-Value | 0.285 |
| ^a^The female partner for the cross. ^b^Pale plants and yellow plants were determined by visual inspection. p > 0.05 considered as significant. | |
